# Effects of Mg and Sb Substitution on the Magnetic Properties of Magnetic Field Annealed MnBi Alloys

**DOI:** 10.3390/nano10112265

**Published:** 2020-11-16

**Authors:** Hui-Dong Qian, Yang Yang, Jung Tae Lim, Jong-Woo Kim, Chul-Jin Choi, Jihoon Park

**Affiliations:** 1 Powder & Ceramics Division, Korea Institute of Materials Science, Changwon 51508, Korea; qianhuidong@kims.re.kr (H.-D.Q.); yangyang@kims.re.kr (Y.Y.); jungtae0401@kims.re.kr (J.T.L.); jwk@kims.re.kr (J.-W.K.); 2 School of Materials Science and Engineering, Pusan National University, Busan 46241, Korea

**Keywords:** MnBi, Mg substitution, Sb substitution, magnetic properties, magnetic field annealing

## Abstract

Rare-earth-free permanent magnets have attracted considerable attention due to their favorable properties and applicability for cost-effective, high-efficiency, and sustainable energy devices. However, the magnetic field annealing process, which enhances the performance of permanent magnets, needs to be optimized for different magnetic fields and phases. Therefore, we investigated the effect of composition on the crystallization of amorphous MnBi to the ferromagnetic low-temperature phase (LTP). The optimal compositions and conditions were applied to magnetic field annealing under 2.5 T for elemental Mg- and Sb/Mg pair-substituted MnBi. The optimum MnBi composition for the highest purity LTP was determined to be Mn_56_Bi_44_, and its maximum energy product, (*BH*)_max_, was 5.62 MGOe. The Mg-substituted MnBi exhibited enhanced squareness (*M*_r_/*M*_s_), coercivity (*H*_c_), and (*BH*)_max_ values up to 0.8, 9659 Oe, and 5.64 MGOe, respectively, whereas the same values for the Sb/Mg pair-substituted MnBi were 0.76, 7038 Oe, and 5.60 MGOe, respectively. The substitution effects were also investigated using first-principles calculations. The density of states and total magnetic moments of Mn_16_Bi_15_Mg and Mn_16_Bi_15_Sb were similar to those of pure Mn_16_Bi_16_. Conversely, the Sb-substituted MnBi resulted in a dramatic enhancement in the anisotropy constant (*K*) from a small negative value (−0.85 MJ/m^3^) to a large positive value (6.042 MJ/m^3^).

## 1. Introduction

Rare-earth-free permanent magnets featuring high maximum energy products ((*BH*)_max_) between those of rare-earth and traditional rare-earth-free permanent magnets are in high demand for cost-effective, high-efficiency, and sustainable energy devices [1,2]. Among rare-earth-free permanent magnetic materials, MnBi exhibits the most promising magnetic properties, featuring high (*BH*)_max_ values that exceed 9 MGOe for powder [3,4,5,6,7] and 7 MGOe for bulk [7,8,9,10]. Notably, the theoretical (*BH*)_max_ of MnBi is 17.7 MGOe at 300 K [11]. During compaction of the powder samples, the reduced coercivity (*H*_c_) degrades the (*BH*)_max_ for bulk MnBi. Consequently, the addition of secondary elements or materials, such as Ga (e.g., Mn_55_Bi_44_Ga) [12], Sn (e.g., MnBi_1-*x*_Sn*_x_*) [13], NaCl or C [14], and nanoparticles [15], to MnBi has been investigated in efforts to increase the *H*_c_. Furthermore, magnetic field annealing has been used to enhance the magnetic properties for permanent magnet applications [16,17,18,19,20,21].

Magnetic field annealing, a process widely used to fabricate Alnico magnets [22], induces aligned texturing in the microstructure. However, the process is not efficient for all permanent magnets owing to variations in the field dependence of the magnetic phase crystallization. Secondary elements such as Sn, Sb, Mg, and In have been substituted/doped to enhance the effectiveness of field annealing for MnBi permanent magnetic materials [23,24,25,26,27]. The magnetic properties of the secondary element-doped/substituted MnBi are superior to those of MnBi fabricated using a field annealing process, revealing higher magnetization, *H*_c_, and squareness (*M*_r_/*M*_s_) than those of binary MnBi [23,24,25,26,27]. Therefore, the highest (*BH*)_max_ (12 MGOe) was achieved for Mn_50_Bi_46_Mg_3_In_0.5_Sb_0.5_ bulk magnetic material [27].

In this study, we systematically investigated the composition (Mn_52+*x*_Bi_48−*x*_, 0 ≤ *x* ≤ 8) and annealing condition dependencies of the purity of the low-temperature phase (LTP). The optimized composition was used in magnetic field annealing experiments under the optimized annealing conditions with an applied field of 2.5 T. The dependence of the magnetic properties on the substitution of Mg and Sb/Mg pairs over large composition ranges, i.e., Mn_56_Bi_44−*x*_Mg_*x*_ (0 ≤ *x* ≤ 5) and Mn_56_Bi_43.5−*x*_Sb_0.5_Mg_*x*_ (0 ≤ *x* ≤ 5), were also systematically investigated. To better comprehend the experimental data, the substitution effects were analyzed using total energies based on different spin configurations, densities of states (DOSs), and magnetic moments.

## 2. Experimental and Calculations

MnBi, Mn-Bi-Mg, and Mn-Bi-Sb-Mg bulks were prepared using the following procedure: melt spinning, cold pressing, and magnetic annealing. The commercial raw materials of Mn (99.95%), Bi (99.999%), Mg (99.98%), and Sb (99.999%) were mixed in the desired atomic ratios of Mn_52+*x*_Bi_48−*x*_ (0 ≤ *x* ≤ 8), Mn_56_Bi_44−*x*_Mg_*x*_ (0 ≤ *x* ≤ 5), and Mn_56_Bi_43.5−*x*_Sb_0.5_Mg*x* (0 ≤ *x* ≤ 5). The mixtures were melted in an Ar environment in quartz nozzles and ejected through a 0.4 mm orifice using pressurized Ar gas onto a copper wheel rotating at a velocity of 40 m/s. The melt-spun ribbons were manually ground (5 min) in ethyl alcohol and then pressed into pellets in tungsten carbide molds (500 MPa). The produced pellets were cut into small pieces (1.5 mm × 1.0 mm × 3 mm) and subjected to magnetic annealing under a magnetic field of 2.5 T. The annealing temperature was optimized by measuring the temperature dependence of the magnetic moment of an amorphous bulk, where the ramping rate was maintained at 16 °C/min. Once the temperature reached the target temperature, the heating element was immediately removed to rapidly cool the samples.

The crystalline structures and phase purities of the products were characterized using X-ray diffraction (XRD) with Cu Kα (λ = 1.5406 Å) radiation (Rigaku Inc., D/Max-2500VL/PC, Tokyo, Japan). Further, the measured XRD patterns were analyzed using the Rietveld refinement method with the program FullProf. Magnetic annealing and magnetic property measurements were performed using a vibrating sample magnetometer (VSM) (Microsense, EZ9, Lowell, MA, USA). The magnetic properties of the bulk samples were corrected according to their shape with a demagnetization factor of 0.1655.

**Figure 1:**
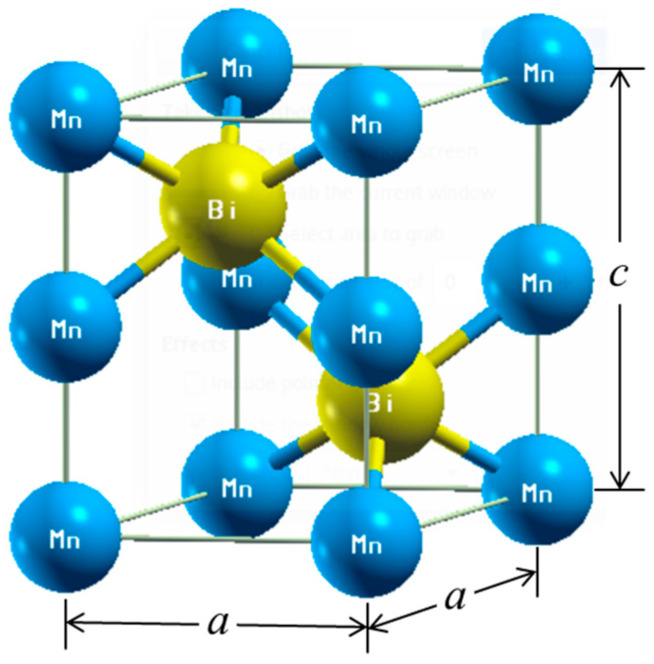
Hexagonal low-temperature phase (LTP) MnBi. The Mn and Bi atoms are blue and yellow, respectively.

The WIEN2k package [28] was used to perform first-principles calculations. The package is based on density functional theory (DFT) and uses the full-potential linearized augmented plane wave method. The 3p, 3d, and 4s states of Mn; 4d, 4f, 5p, 5d, 6s, and 6p states of Bi; 3s state of Mg; and 4p, 4d, 5s, and 5p states of Sb were treated as valence states in the calculations. Unit cell and supercell calculations used 28 × 28 × 24 and 25 × 25 × 15 reciprocal space meshes, which generate 18,816 and 9375 k-points in the irreducible part of the Brillouin zone, respectively. The total energies were calculated within an accuracy of 0.0013 eV. The experimental lattice constants (*a* = 4.256 Å and *c* = 6.042 Å at 4.2 K) [29] in Figure 1 were used, and the muffin-tin radii (RMT) for Mn, Bi, Mg, and Sb were set as 2.5 a.u. The cutoff parameter of PMT × *K*_max_ = 7.0 with *l*_max_ = 10 inside the muffin-tin spheres was used to expand the wavefunctions in terms of the lattice harmonics. All spin-polarized and spin-orbit coupling calculations were based on DFT within the local-spin-density approximation (LSDA).

## 3. Results and Discussion

**Figure 2:**
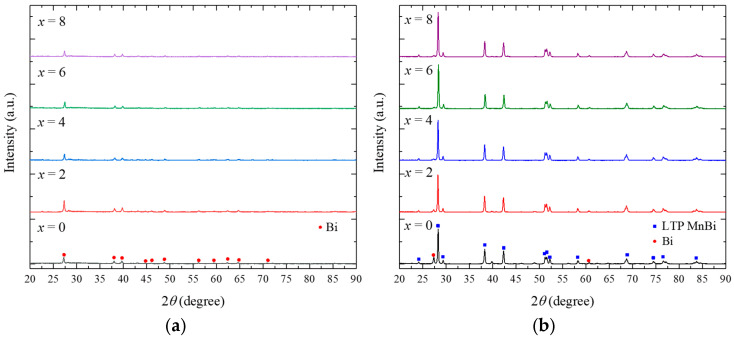
XRD patterns of the (**a**) ground Mn_52+*x*_Bi_48__−*x*_ melt-spun ribbons and (**b**) annealed bulk samples (300 °C for 24 h).

The degree of amorphization plays an important role in crystallizing LTP Mn_50_Bi_50_ in the direction of the magnetic field. Therefore, we prepared Mn_52+*x*_Bi_48__−_*_x_* (0 ≤ *x* ≤ 8) samples to investigate the composition dependence of the degree of amorphization and their crystallization to LTP Mn_50_Bi_50_ after annealing at 300 °C for 24 h. Melt-spun Mn_52+*x*_Bi_48__−_*_x_* (0 ≤ *x* ≤ 8) products contain small amounts of droplet-shaped particles and ribbons. These particles were removed, and only the ribbons were collected to fabricate bulk samples. It is noted that Mn and Bi were partially reacted with quartz nozzle during the melt-spinning process; therefore, the final compositions converge into Mn_50_Bi_50_ regardless of the initial compositions. The bulks were loaded into a vacuum tube furnace and annealed. Figure 2a shows the XRD patterns of the Mn_52+*x*_Bi_48__−_*_x_* (0 ≤ *x* ≤ 8) melt-spun ribbons. All the samples exhibited minor peaks of Bi, whereas LTP Mn_50_Bi_50_ was not detected. Therefore, the melt-spun ribbons mostly comprised an amorphous phase with a small quantity of Bi crystallites. The differences in the peak intensities of Bi for the five samples were indistinguishable, except for Mn_54_Bi_46_. Figure 2b shows the XRD patterns of the annealed Mn_52+*x*_Bi_48__−_*_x_* (0 ≤ *x* ≤ 8) bulk samples. The purities of the samples were initially enhanced with increasing *x*, but they were not enhanced significantly when *x* ≥ 4; the calculated purities were 84%, 93%, 96%, 96%, and 97% for *x* = 0, 2, 4, 6, and 8, respectively. Therefore, we used the composition *x* = 4, i.e., Mn_56_Bi_44_, for the following experiment.

**Figure 3:**
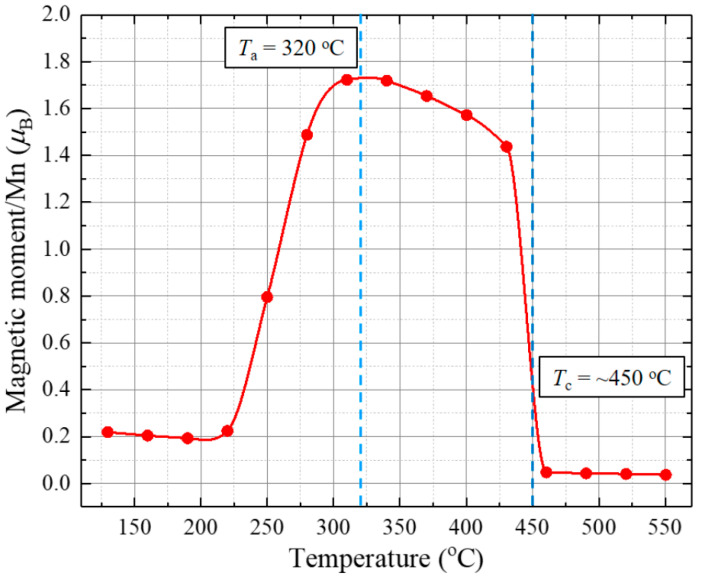
Temperature dependence of the magnetic moment per Mn atom of an amorphous Mn_56_Bi_44_ bulk sample under an applied magnetic field of 2.5 T.

The annealing temperature of amorphous Mn_56_Bi_44_ was optimized using VSM. Figure 3 depicts the temperature dependence of the magnetic moment per Mn atom of an amorphous Mn_56_Bi_44_ bulk sample under an applied magnetic field of 2.5 T. The amorphous Mn_56_Bi_44_ bulk sample was heated from 130 to 550 °C while its magnetic moment was measured using VSM. The measured magnetic moment of the paramagnetic amorphous bulk sample initially decreased with increasing temperature but began increasing at 220 °C due to crystallization of the amorphous phase. The highest magnetic moment was measured at temperatures ranging from 310 to 340 °C. It is noted that further heating of the sample above 355 °C results in irreversible decomposition of LTP Mn_50_Bi_50_ into Mn_52_Bi_48_ + Bi [30]. Therefore, we selected an annealing temperature of 320 °C for the annealing process. Notably, the magnetic moment declined at temperatures between 430 and 460 °C, with a sharp decrease at 450 °C. This temperature is considerably higher than the phase transformation temperature (340–360 °C) of MnBi from LTP to the high-temperature phase (HTP) [29]. This temperature disagreement at high temperatures may be attributed to heat loss in the open heating circuit of the VSM system.

**Figure 4:**
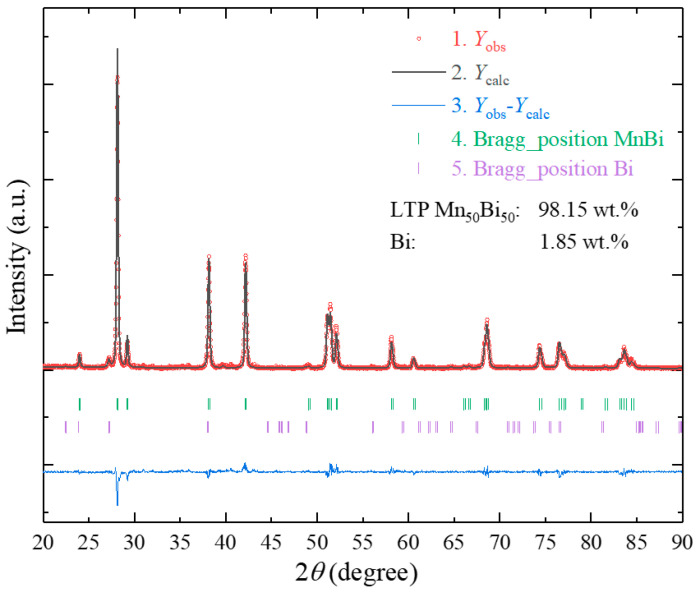
Refined XRD pattern of the annealed Mn_56_Bi_44_ bulk samples (320 °C for 5 min).

Figure 4 depicts the refined XRD pattern of the Mn_56_Bi_44_ bulk sample annealed at 320 °C for 5 min. The phase identity and weight fraction were analyzed using the Rietveld refinement method with the program FullProf. The refined XRD pattern confirmed that the annealed Mn_56_Bi_44_ bulk had a hexagonal structure with a space group of *P*63/*mmc*, and the lattice constants *a*_0_ and *c*_0_ were 4.285 ± 0.001 and 6.113 ± 0.001 Å, respectively. The annealed Mn_56_Bi_44_ bulk mainly comprised the LTP, and the peak at approximately 27.24° corresponds to an impure Bi phase. After heat treatment at 320 °C for 5 min, the purity of the LTP Mn_50_Bi_50_ increased to 98.15 wt.%. The Mn and Bi atoms occupied the 2a site (0, 0, 0) and the 2c site (1/3, 2/3, 1/4), respectively [31].

**Figure 5:**
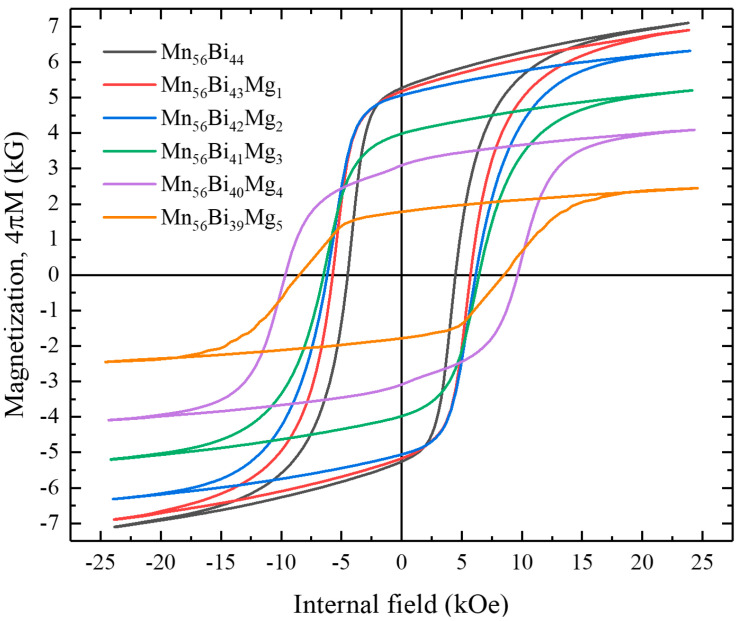
Magnetic hysteresis loops of Mn_56_Bi_44__−*x*_Mg*_x_* (0 ≤ *x* ≤ 5) bulk samples.

**Figure 6:**
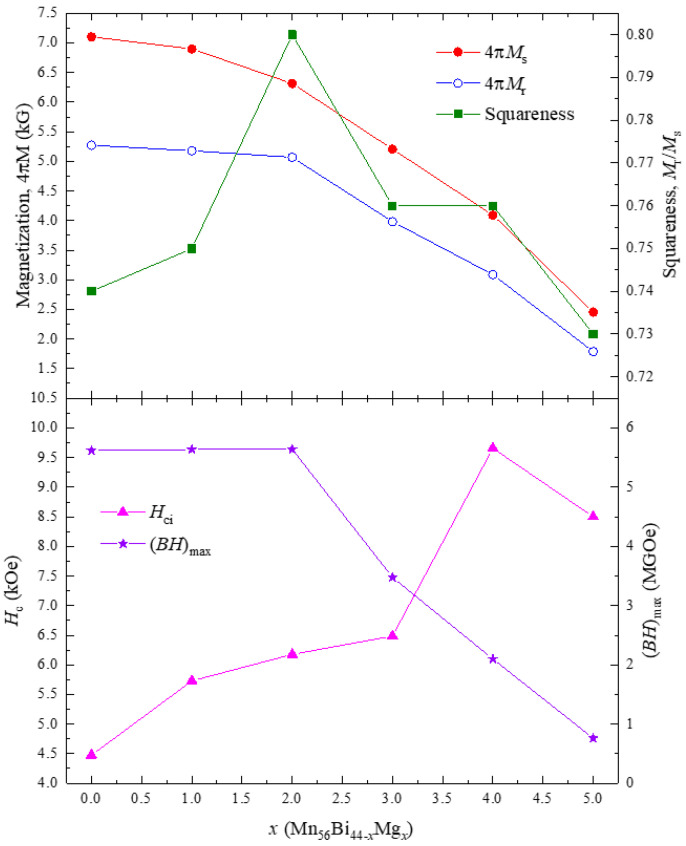
Composition dependence of magnetization, squareness, *H*_c_, and (*BH*)_max_ for the Mn_56_Bi_44-*x*_Mg*_x_* (0 ≤ *x* ≤ 5) bulk samples.

The magnetic hysteresis loops of the Mn_56_Bi_44__−_*_x_*Mg*_x_* (0 ≤ *x* ≤ 5) bulk samples obtained after magnetic annealing under 2.5 T are shown in Figure 5. The hysteresis loops were smooth and did not contain any kinks until *x* = 3; however, a kink appeared at *x* = 4. As the Mg content increased, the magnetization at the maximum magnetic field and remanent magnetization decreased, whereas *H*_c_ increased to 9659 Oe for *x* = 4 and then decreased at higher values of *x*. The magnetization, squareness, *H*_c_, and (*BH*)_max_ of the Mn_56_Bi_44__−_*_x_*Mg*_x_* (0 ≤ *x* ≤ 5) bulk samples are plotted in Figure 6. Although the magnetization at the maximum field and remanent magnetization decreased as *x* increased, (*BH*)_max_ increased slightly from 5.62 MGOe (for Mn_56_Bi_44_) to 5.64 MGOe (for Mn_56_Bi_43_Mg_1_ and Mn_56_Bi_42_Mg_2_) because of the increased squareness (*M*_r_/*M*_s_ ≈ 0.8). At *x* values exceeding 2, (*BH*)_max_ decreased linearly. The decreasing magnetization and increasing *H*_c_ for *x* ≥ 2 originate from phase decomposition into nonmagnetic phases. The XRD patterns for the field-annealed samples will be published elsewhere. These impurities act as pinning centers for domain walls by strongly inhibiting their motion, thus reducing the efficiency of this reversal mechanism.

**Figure 7:**
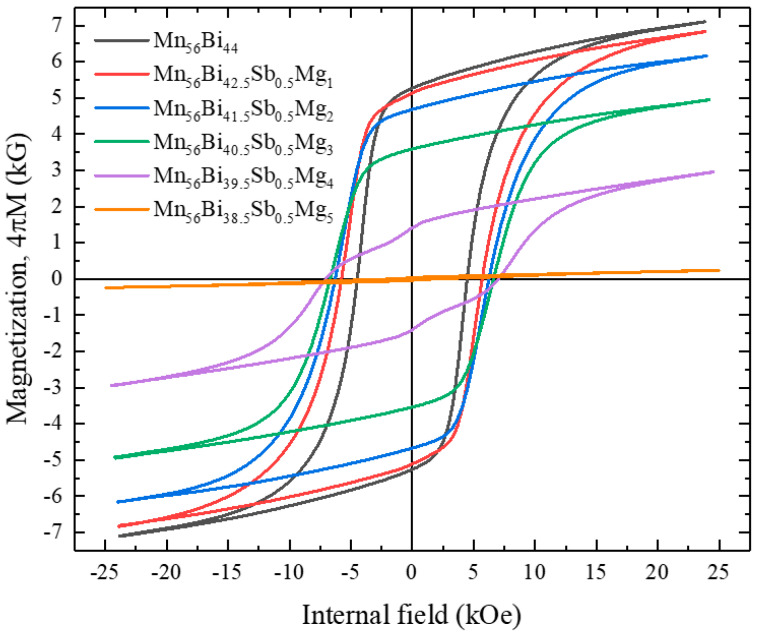
Magnetic hysteresis loops of Mn_56_Bi_43.5__−*x*_Sb_0.5_Mg*_x_* (0 ≤ *x* ≤ 5) bulk samples.

**Figure 8:**
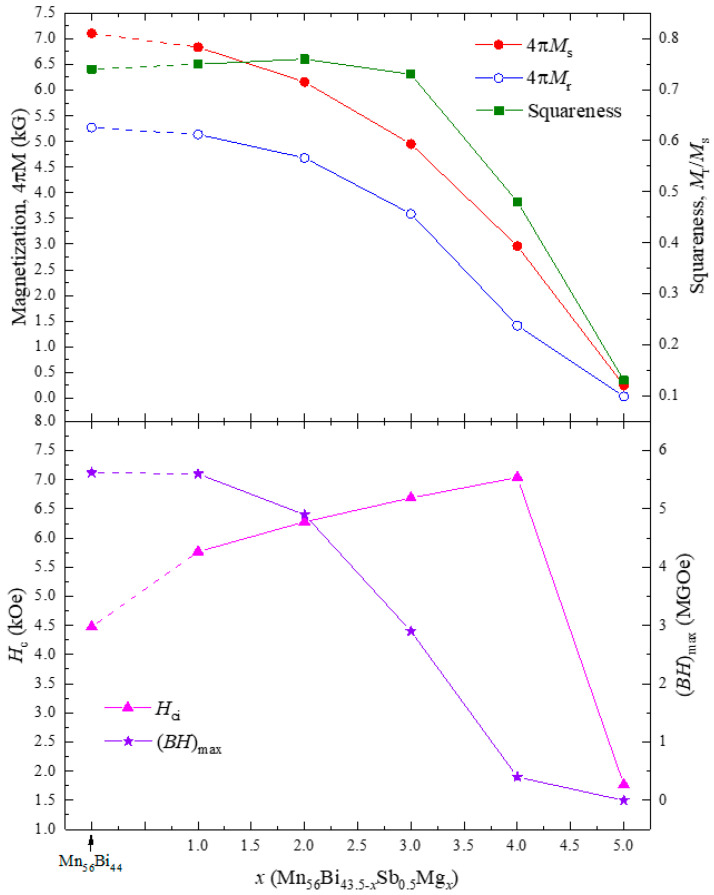
Composition dependence of magnetization, squareness, *H*_c_, and (*BH*)_max_ for Mn_56_Bi_43.5__−*x*_Sb_0.5_Mg*_x_* (0 ≤ *x* ≤ 5) bulk samples.

Sb-substituted Mn-Bi-Mg, i.e., Mn_56_Bi_43.5__−_*_x_*Sb_0.5_Mg*_x_* (0 ≤ *x* ≤ 5) bulk, was also fabricated to investigate the effect of Sb/Mg pair substitution on the magnetic properties. The magnetic hysteresis loops of the Mn_56_Bi_43.5__−_*_x_*Sb_0.5_Mg*_x_* (0 ≤ *x* ≤ 5) bulk samples are shown in Figure 7. The magnetic annealing process and conditions were the same as those for Mn_56_Bi_44__−_*_x_*Mg*_x_* (0 ≤ *x* ≤ 5). The magnetization at maximum field and remanent magnetization decreased with increasing Mg content, while *H*_c_ linearly increased until *x* = 4, reaching 7038 Oe, as shown in Figure 8. The hysteresis loops were smooth, containing no kinks until *x* = 3, but a kink appeared at *x* = 4. Substitution of a small amount of Sb into Mn-Bi-Mg increased *H*_c_ until *x* = 3 but caused severe phase decomposition at higher Mg contents; the LTP was completely decomposed at *x* = 5. The increased (*BH*)_max_ for Mn_56_Bi_44__−_*_x_*Mg*_x_* (0 ≤ *x* ≤ 5) is attributed to the enhanced squareness. However, the squareness monotonically decreased with increasing Mg content for Mn_56_Bi_43.5__−_*_x_*Sb_0.5_Mg*_x_* (0 ≤ *x* ≤ 5), resulting in decreased (*BH*)_max_. The decreasing magnetization and increasing *H*_c_ for *x* ≥ 3 are attributed to the existence of nonmagnetic secondary phases, which act as pinning centers for the domain walls.

**Figure 9:**
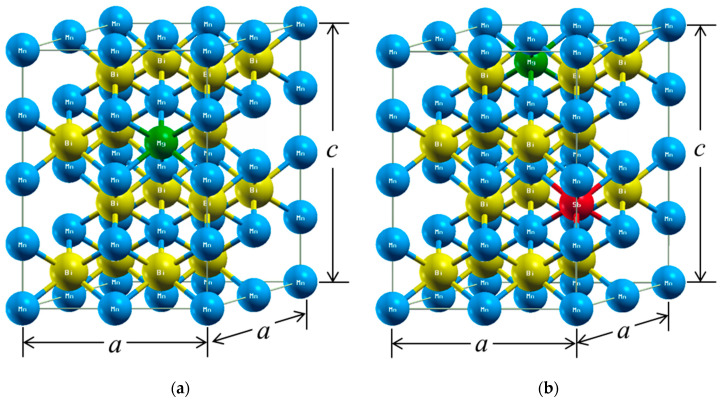
Crystal structures of (**a**) Mn_16_Bi_15_Mg (or Sb) and (**b**) Mn_16_Bi_14_SbMg used for the first-principles calculations. The Mn, Bi, Mg, and Sb atoms are in blue, yellow, green, and red, respectively.

**Figure 10:**
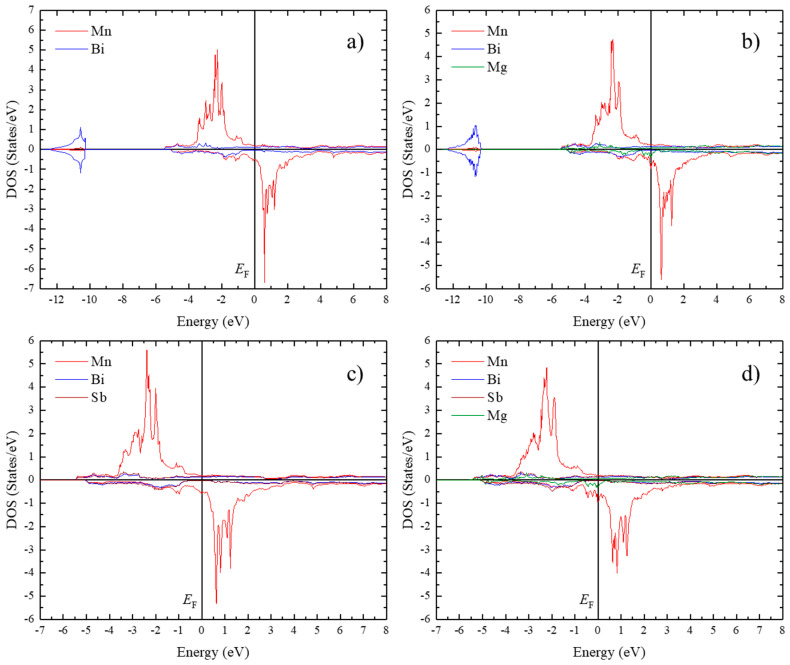
Density of states (DOS) for (**a**) MnBi, (**b**) Mn_16_Bi_15_Mg, (**c**) Mn_16_Bi_15_Sb, and (**d**) Mn_16_Bi_14_SbMg.

The substitution effects of Mg and Sb on the magnetic properties of MnBi were also investigated using first-principles calculations. Mg and Sb are presumed to substitute in the Bi site in the unit cell of MnBi (Figure 1). The crystal structures of Mn_16_Bi_15_Mg (or Sb) and Mn_16_Bi_14_SbMg for the calculations are shown in Figure 9. Figure 10 shows the DOSs of LTP MnBi, Mn_16_Bi_15_Mg, Mn_16_Bi_15_Sb, and Mn_16_Bi_14_SbMg, which were calculated based on spin-polarized and spin-orbit coupling. The *s* bands of Bi, Mg, and Sb mainly contributed to the low-energy region below the Fermi energy (*E*_F_), whereas the *d* band of Mn mainly contributed to the high-energy region above *E*_F_, as seen in Figure 10. The DOS is a highly degenerate energy state that is slightly below and above *E*_F_ in the majority and minority spin states, respectively. These highly degenerate energy states near the *E*_F_ are the origin of the magnetic moment of LTP MnBi-based magnetic materials because they contribute to the difference between the number of electrons in the majority and minority spin states below the *E*_F_. In contrast to the highly degenerate DOSs below (above) the *E*_F_ for the majority (minority) spin states of LTP MnBi, Mn_16_Bi_15_Mg, Mn_16_Bi_15_Sb, and Mn_16_Bi_14_SbMg (see Figure 10), the *E*_F_ lies at the low DOS between the highly degenerated spin states for the majority and minority spin states. This *E*_F_ position is the origin of the phase stability of the above-mentioned LTP MnBi-based magnetic materials.

The spin magnetic moments for Mn, Bi, Mg, and Sb and the anisotropy constants (*K*) for MnBi, MnMg, Mn_16_Bi_15_Mg, Mn_16_Bi_15_Sb, and Mn_16_Bi_14_SbMg are given in Table 1. The spins of the Mn atoms in MnMg were antiparallel to each other, resulting in a small total magnetic moment. The magnetic moment of Mn was approximately 3.57 *μ*_B_, whereas those of Bi, Mg, and Sb were approximately −0.1 *μ*_B_ for all ferromagnetic MnBi-based materials, as shown in Table 1. Therefore, the magnetic properties of MnBi-based magnetic materials are mostly dependent on the Mn atoms. The total magnetic moments of a Mg or Sb element or a Mg/Sb pair-substituted MnBi were not degraded; the total magnetic moments of Mn_16_Bi_15_Mg, Mn_16_Bi_15_Sb, and Mn_16_Bi_14_SbMg were similar to that of MnBi (56.352 *μ*_B_ = 7.044 *μ*_B_ × 8 unit cells).

The *K* value of MnBi was calculated to be −0.850 × 10^6^ J/m^3^, which corresponds to an in-plane spin direction. The spins of MnBi at 0 K lie in the basal plane but rotate to the *c*-axis at approximately 90 K [9]. The *K* of Mg-substituted MnBi, i.e., Mn_16_Bi_15_Mg, was almost identical to that of pure MnBi, whereas that of Sb-substituted MnBi, i.e., Mn_16_Bi_15_Sb, was much larger than Mn_16_Bi_15_Mg in the *c*-axis, as shown in Table 1. Therefore, one Mg element substitution into Mn_16_Bi_16_ resulted in no *K* change, whereas one Sb element substitution resulted in an out-of-plane spin direction; this property is necessary for the development of permanent magnets. Moreover, the *K* of 6.042 × 10^6^ J/m^3^ for Mn_16_Bi_15_Sb is comparable to that of commercial rare-earth permanent magnets, such as 4.9 × 10^6^ J/m^3^ for Nd_2_Fe_14_B and 4.2 × 10^6^ J/m^3^ for Sm_2_Co_17_ [32]. In addition, the *K* of the Sb/Mg pair-substituted MnBi, i.e., Mn_16_Bi_14_SbMg, was larger than that of Mn_16_Bi_15_Mg due to the substitution effect of Sb.

## 4. Conclusions

We optimized the initial Mn-Bi composition and annealing temperature for high-purity LTP using melt-spinning and annealing processes. The optimized annealing conditions were applied to fabricate Mg and Mg- and Sb-substituted Mn-Bi using magnetic annealing. The magnetic field annealing of Mn_56_Bi_44_ resulted in an enhanced squareness of 0.74, which was further increased to 0.8 upon Mg substitution. The highest coercivity and maximum energy product of Mg-substituted Mn-Bi were 9659 Oe (for Mn_56_Bi_40_Mg_4_) and 5.64 MGOe (for Mn_56_Bi_42_Mg_2_), respectively. First-principles calculations indicated that the total magnetic moments of Mn_16_Bi_15_Mg and Mn_16_Bi_15_Sb were similar to the total magnetic moment of pure Mn_16_Bi_16_. In contrast, the anisotropy constant *K* changed from −0.85 to 6.042 MJ/m^3^ when Sb was substituted, indicating that the spin direction in the basal plane changed to the *c*-axis.

## Figures and Tables

**Table 1:** Calculated Mn, Bi, Mg, Sb, interstitial, and total magnetic moments and anisotropy constants.

Materials	Mn	Bi	Mg	Sb	Interstitial, Int.	Total Magnetic Moments, *μ*_B_	Anisotropy Constant, *K* (×10^6^ J/m^3^)
MnBi	3.571	−0.103	-	-	0.104	7.044	−0.850
MnMg	2.331	-	0.002	-	0.006	0.067	19.690
Mn_16_Bi_15_Mg	3.577	−0.108	−0.071	-	0.790	56.371	−0.898
Mn_16_Bi_15_Sb	3.573	−0.121	-	−0.124	0.741	56.210	6.042
Mn_16_Bi_14_SbMg	3.579	−0.134	−0.071	−0.131	0.784	56.368	−0.441
